# Asymmetric scaling of cerebellar cortex and deep nuclei reshapes input-output architecture across primates

**DOI:** 10.21203/rs.3.rs-9464995/v1

**Published:** 2026-04-28

**Authors:** Kadharbatcha S Saleem, Alexandru V Avram, Daniel Glen, Peter J Basser

**Affiliations:** 1 Section on Quantitative Imaging and Tissue Sciences (SQITS), Eunice Kennedy Shriver National Institute of Child Health and Human Development (NICHD), NIH, Bethesda, MD 20892;; 2 Scientific and Statistical Computing Core, National Institute of Mental Health (NIMH), NIH, Bethesda, MD 20892.

## Abstract

The cerebellum shapes distributed motor and association networks through precisely organized pathways linking the cerebellar cortex to the deep cerebellar nuclei (DCN), its principal output structures. Whether these cortical and nuclear compartments scale proportionally across primates, and how their relative expansion relates to cerebellar organization, remains unresolved. Here, we generate multimodal, high-resolution cross-species 3D cerebellar atlases in marmoset, macaque, and human by integrating iron-sensitive and diffusion MRI (MAP-MRI) with histological markers, enabling direct delineation of cerebellar lobules and DCN subdivisions within a unified framework. Comparative volumetric analyses reveal strikingly nonuniform scaling of cerebellar cortical-nuclear architecture: cortical expansion markedly outpaces DCN enlargement, indicating disproportionate growth of input relative to output systems. This divergence is accompanied by progressive reorganization of the DCN, with increasing dominance of the dentate nucleus. In contrast, cortical expansion is driven by posterior hemispheric territories, with lobules VI-IX and Crus I/II, linked to higher-order functions, showing the strongest scaling and largest absolute gains, whereas DCN subdivisions show selective rather than uniform scaling. Together, these findings establish nonuniform cortical-nuclear scaling as a systems-level organizational principle that reshapes cerebellar input-output architecture across primates.

## Introduction

The cerebellum is increasingly recognized as a central hub within distributed brain systems supporting sensorimotor, cognitive, and affective functions^1–4^. Rather than serving solely motor coordination, it modulates widespread cortical and subcortical networks through precisely organized cortico-ponto-cerebellar-nuclear pathways^5,6^. Within this architecture, the cerebellar cortex performs extensive local computations, whereas the deep cerebellar nuclei (DCN) constitute the sole output channels of cerebellar processing^7,8^. Consequently, the relative scaling of cortical input territories and nuclear output structures imposes a fundamental constraint on how cerebellar computations are transmitted to the rest of the brain.

Comparative studies in primates have revealed pronounced cerebellar expansion, particularly within posterior hemispheric territories linked to association cortex^9–11^. Posterior lobules, including lobules VI-IX and the ansiform lobule (Crus I/II) are disproportionately enlarged in anthropoid primates and are consistently engaged during higher-order cognitive tasks in humans^1,12^. These observations have reinforced the view that the cerebellum contributes to distributed cognitive networks beyond motor control. However, it remains unclear whether expansion of the cerebellar cortical sheet is matched by proportional scaling of the DCN. Because the DCN form the exclusive output interface of the cerebellum, any imbalance between cortical and nuclear scaling would imply a fundamental reorganization of cerebellar input–output architecture across evolution.

The DCN are subdivided into dentate, interposed, and fastigial nuclei, each with distinct connectivity and functional roles^7,8^. In particular, the dentate nucleus (DN) is implicated in closed-loop circuits linking the lateral cerebellum with prefrontal, parietal, and other association cortices via thalamic relays^6,13–16^. While the DN is thought to support cognitive and motor planning functions, the degree to which it expands relative to other nuclei across primates remains unresolved. In parallel, the interposed and fastigial nuclei are more closely associated with sensorimotor and axial control systems, suggesting that differential scaling across DCN subregions may reflect functional specialization.

Despite extensive work on cerebellar morphology, quantitative cross-species comparisons of DCN subdivisions remain limited, largely due to challenges in resolving nuclear boundaries with conventional MRI. Iron-sensitive MRI provides additional contrast related to cytoarchitecture and iron accumulation^17–19^, but its cross-species interpretability has not been fully established. Moreover, existing atlases are typically species-specific, limiting direct structural comparison across primates.

Addressing these limitations requires multimodal, anatomically precise approaches that integrate MRI with histological validation. High-resolution imaging combined with cytoarchitectonic reference data enables consistent delineation of cerebellar cortical and nuclear compartments across species. In particular, histology-constrained segmentation and iron-sensitive contrasts provide complementary information for defining DCN subregions, while ultra-high-resolution histological datasets such as BigBrain^20^ enable translation of postmortem anatomy into in vivo human imaging space.

Here, we generate multimodal, high-resolution cerebellar atlases in marmoset, macaque, and human by integrating structural MRI, iron-sensitive imaging, and histological validation of both cerebellar cortex and DCN. This framework establishes a common anatomical reference system for cross-species comparison and enables quantitative assessment of cerebellar scaling across hierarchical levels. We first establish a histology-validated DCN segmentation framework and characterize species-dependent differences in nuclear microstructure and MRI contrast. We then quantify volumetric scaling across DCN subregions and evaluate whether cerebellar output structures scale proportionally with overall cerebellar size. Finally, we assess cortical-lobular organization, with emphasis on posterior hemispheric territories and Crus I/II, to determine how input and output compartments are differentially reorganized across primates.

Together, this hierarchical approach reveals nonuniform scaling between cerebellar cortical input and nuclear output compartments across primates. The posterior cerebellar cortex expands disproportionately relative to the deep cerebellar nuclei (DCN), which scale sublinearly and show increasing dentate dominance. These findings identify nonuniform cortical-nuclear scaling as a systems-level organizational principle that underlies asymmetric cerebellar expansion across primates.

## RESULTS

### Multimodal MRI-histology atlases enable cross-species cerebellar comparison

We integrated high-resolution structural and mean apparent propagator (MAP) MRI, iron-sensitive imaging, and histological validation to generate cross-species cerebellar atlases in marmoset, macaque, and human. This framework enables direct quantitative comparison of cerebellar lobular territories and deep cerebellar nuclei (DCN) across primates. DCN subdivisions were delineated using convergent MRI contrasts, including iron-sensitive and immunohistochemical markers, revealing conserved topology alongside species-specific differentiation.

Volumetric analyses show that cerebellar cortical expansion exceeds DCN enlargement across primates, indicating disproportionate scaling of input relative to output compartments. This dissociation is accompanied by progressive expansion of posterior hemispheric territories, particularly lobules VI-IX and Crus I/II, whereas DCN exhibit selective rather than uniform scaling across subregions. Together, these findings demonstrate nonuniform scaling of cerebellar input-output architecture across primates.

### Species-dependent iron patterns shape DCN MRI contrast

**Fig. 1 |:**
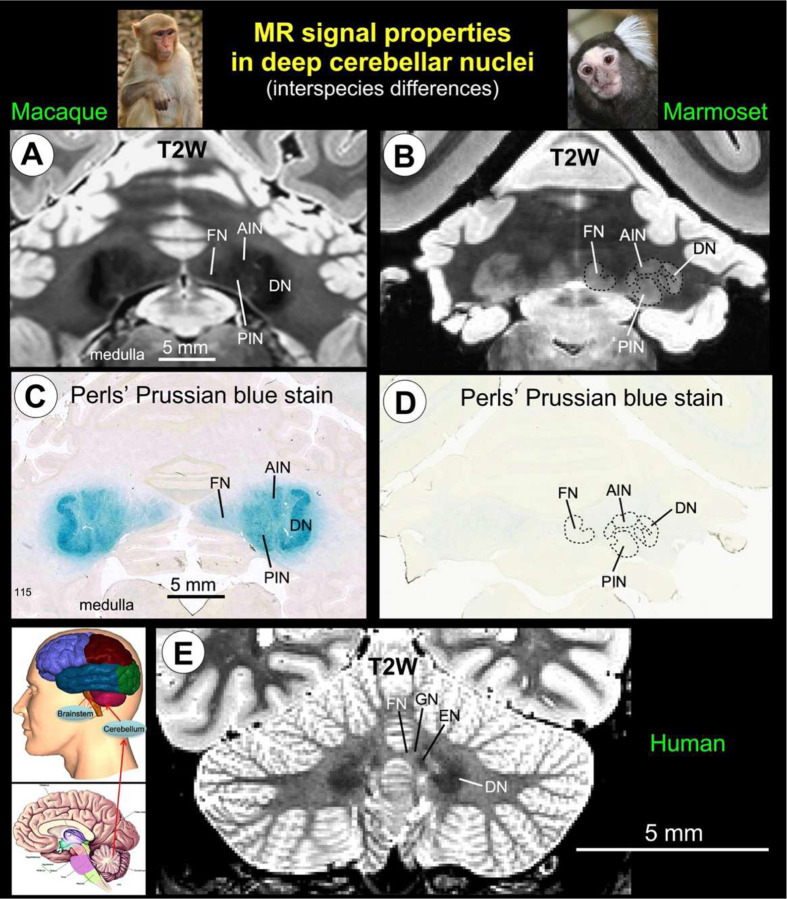
Iron-sensitive MRI contrast enables cross-species identification of the DCN. **A-B**, High-resolution T2-weighted MRI of the deep cerebellar nuclei (DCN) in macaque and marmoset, showing the dentate nucleus (DN), anterior interposed nucleus (AIN/emboliform nucleus, EN), posterior interposed nucleus (PIN/globose nucleus, GN), and fastigial nucleus (FN). Marked species differences in signal intensity are observed, with pronounced hypointensity in macaque DCN and reduced or inverted contrast in marmoset. **C-D**, Corresponding histological sections stained with Perls’ Prussian blue for ferric iron. Macaque DCN show strong iron labeling across nuclear territories, whereas marmoset DCN exhibit minimal iron accumulation, consistent with MRI contrast differences. **E**, In vivo human T2-weighted MRI (Human Connectome 1.0 Project dataset) showing DCN hypointensity comparable to macaque, indicating conserved iron-associated contrast in anthropoid primates. Together, these data demonstrate a tight correspondence between MRI signal intensity and histologically measured iron content across species, establishing iron-dependent contrast as a conserved biophysical marker for DCN delineation and cross-species cerebellar mapping.

Using high-resolution MRI with histological validation, we examined whether interspecies differences in deep cerebellar nuclei (DCN) signal reflect variation in underlying tissue composition, with a focus on iron-sensitive contrast (Fig. 1). Across primates, DCN exhibited marked differences in T2-weighted signal. In macaques and humans, the DCN appeared strongly hypointense relative to surrounding cerebellar tissue, whereas marmosets showed reduced or reversed contrast patterns (Fig. 1A, B, E). Histological analysis using Perls’ Prussian blue staining confirmed these differences. Macaque DCN showed robust iron labeling throughout the neuropil, whereas marmoset DCN exhibited minimal staining, indicating substantially lower iron content (Fig. 1C, D). Together, these findings demonstrate species-dependent variation in cerebellar iron content, with elevated iron-associated contrast in anthropoid primates relative to marmosets, providing a basis for interpreting cross-species differences in MRI signal.

### Histology-validated mapping of deep cerebellar nuclei across primates

**Fig 2 |:**
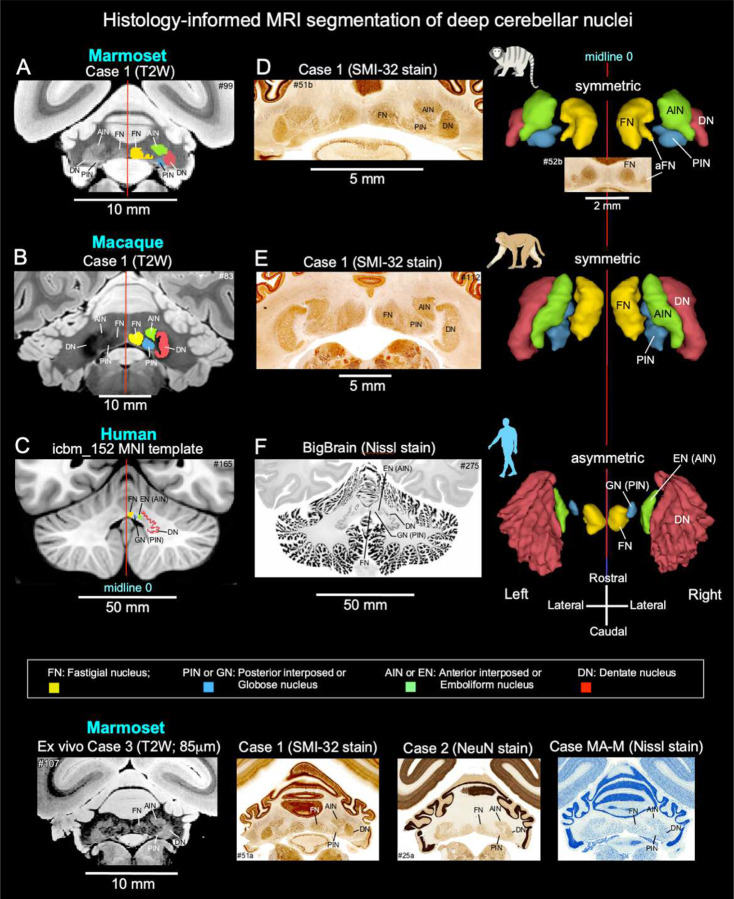
Histology-guided segmentation establishes a cross-species framework for deep cerebellar nuclei delineation **A-C**, Delineation of deep cerebellar nuclei (DCN): dentate nucleus (DN), anterior interposed nucleus (AIN/emboliform nucleus, EN), posterior interposed nucleus (PIN/globose nucleus, GN), and fastigial nucleus (FN), in high-resolution T2-weighted MRI of marmoset (150 μm; case 1), macaque (200 μm; case 1), and human (MNI template, 500 μm). Despite differences in brain size and resolution, conserved nuclear topology is observed across species. **D-E**, MRI-defined DCN boundaries are validated against matched histological sections stained with SMI-32, demonstrating close correspondence in nuclear borders and internal cytoarchitectonic organization. In marmosets, a cell-sparse boundary and strong neuropil differentiation support clear DN-interposed separation; in macaques, conserved cell-sparse zones confirm homologous boundaries despite more uniform labeling intensity. **F**, In humans, BigBrain-derived histological reconstructions registered to MNI space enable precise delineation of DCN subregions, including the highly folded dentate nucleus, which is not reliably resolved using conventional T1-weighted templates alone. **Right column**, Three-dimensional reconstructions of DCN across species (superior view) illustrate conserved rostrocaudal organization alongside species-specific morphological variation. In marmosets, a small accessory fastigial nucleus (aFN) is consistently identified adjacent to the fastigial nucleus (inset: SMI-32 staining). **Bottom row**, Independent validation using a high-resolution marmoset dataset (85 μm isotropic T2weighted imaging) confirms reproducibility of DCN segmentation. A distinct cell-sparse boundary separating DN and AIN is resolved in vivo, consistent with histological markers (SMI-32, NeuN, Nissl) from independent specimens. Across species and modalities, MRI-defined DCN subregions show strong correspondence with histological cytoarchitecture in spatial location, morphology, and internal organization, establishing a validated framework for quantitative cross-species comparison of cerebellar output structures.

The deep cerebellar nuclei (DCN): dentate (DN), anterior interposed (AIN; or emboliform, EN), posterior interposed (PIN; or globose, GN), and fastigial (FN), were delineated in marmosets and macaques using multimodal MRI (T2-weighted, magnetization transfer ratio [MTR], and MAP-MRI) with matched histological validation (SMI-32, NeuN, AChE, Nissl; Fig. 2, Supplementary Fig. 1). At 150–200 μm isotropic resolution, T2-weighted imaging resolved major nuclear boundaries (DN, interposed nuclei, FN), capturing core DCN compartmental organization (Fig. 2A, B; Supplementary Figs. 2, 3). In marmosets, the DN showed partial continuity with the AIN at select rostrocaudal levels (Supplementary Fig. 2, section #96).

Microstructural contrasts varied across species. In marmosets, MTR- and MAP-MRI-derived metrics (radial diffusivity [RD] and return-to-axis probability [RTAP]) enhanced intra-DCN differentiation. In macaques, MTR preserved nuclear contrast, whereas diffusion-derived metrics provided limited additional separation (Supplementary Fig. 1), indicating species-dependent sensitivity of microstructural MRI contrasts.

Histology confirmed MRI-based parcellation and refined nuclear boundaries (Fig. 2D, E; Supplementary Figs. 2, 3). SMI-32 reliably distinguished DN from interposed nuclei in both species. In marmosets, strong DN neuropil labeling and a pronounced cell-sparse boundary zone enabled clear separation from AIN and PIN. In macaques, a comparable cell-sparse interface between DN and interposed nuclei supported segmentation despite more homogeneous labeling. These results indicate that DCN boundaries are consistently defined by microanatomical discontinuities across species. A small accessory fastigial nucleus (aFN), contiguous with FN, was consistently identified in marmosets (Fig. 2, right column; inset). Three-dimensional MRI-histology reconstructions further revealed rostrocaudal organization and species-specific differences in relative expansion, folding, and spatial adjacency of DCN subregions (Fig. 2, right column).

In one marmoset (case 3), high-resolution T2-weighted imaging (85 μm isotropic) resolved a narrow cell-sparse boundary between DN and adjacent nuclei. Although histology was not available for this specimen, the observed pattern matched independent SMI-32, NeuN, and Nissl datasets, supporting the robustness of MRI-defined boundaries.

**Fig. 3 |:**
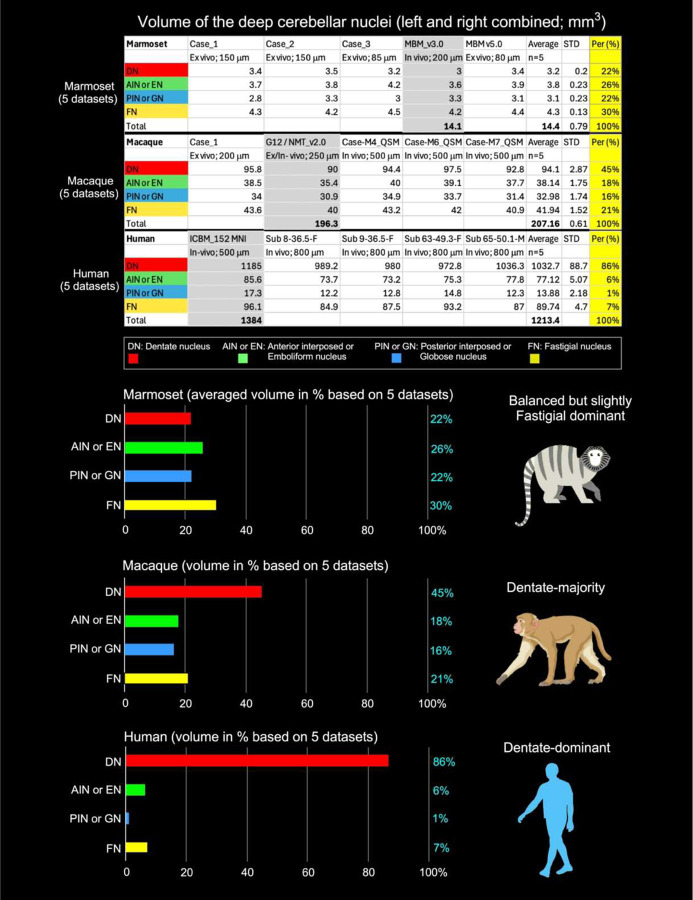
Nonuniform scaling and compositional reorganization of deep cerebellar nuclei across primates **Top**, Absolute volumes of deep cerebellar nuclei (DCN) subregions (bilateral) in marmosets, macaques, and humans, averaged across five individuals per species. Subregions include the dentate nucleus (DN), anterior interposed nucleus (AIN/emboliform nucleus, EN), posterior interposed nucleus (PIN/globose nucleus, GN), and fastigial nucleus (FN). Volumes were derived from histology-informed segmentation, enabling consistent cross-species delineation (Fig. 2). **Bottom**, Relative proportions of DCN subregions expressed as percentage of total DCN volume. A progressive shift toward dentate dominance is observed from marmoset to human, with corresponding reductions in interposed and fastigial nuclei. Together, these data demonstrate nonuniform compositional reorganization of cerebellar output structures across primates.

In humans, DCN subregions were delineated in MNI space (500 μm) using cytoarchitectonic reconstructions from the BigBrain dataset^20^ (Fig. 2C, F; Supplementary Fig. 4). Standard T1-weighted templates did not reliably resolve nuclear boundaries, whereas BigBrain-informed priors enabled identification of all DCN subdivisions, including the folded DN, while preserving rostrocaudal organization. To extend this framework to in vivo data, additional human T2-weighted datasets (800 μm) were segmented using BigBrain-derived priors (Fig. 3, top; Supplementary Fig. 5), enabling consistent DCN delineation across individuals and supporting cross-species volumetric comparisons.

### Scaling of deep cerebellar nuclei across primates

We quantified the relative volumes of deep cerebellar nuclei (DCN) subregions in marmosets, macaques, and humans, averaging measurements from five individuals per species (Fig. 3, top). To enable direct cross-species comparison despite marked differences in absolute brain size, volumes are expressed as percentages of total DCN volume (Fig. 3, bottom).

In marmosets, DCN subregions were relatively evenly distributed. The fastigial nucleus (FN) represented the largest fraction of the DCN (30%), followed by the anterior interposed/emboliform nucleus (AIN/EN; 26%). The dentate nucleus (DN) and posterior interposed/globose nucleus (PIN/GN) contributed similar proportions (~22% each). In macaques, the DN showed a notable relative expansion, accounting for 45% of total DCN volume, while FN contributed 21%, and the AIN/EN and PIN/GN comprised 18% and 16%, respectively. Compared with marmosets, macaques displayed a marked increase in the proportional size of the DN and a corresponding reduction of the interposed nuclei.

In humans, the DCN were overwhelmingly dominated by the dentate nucleus (DN, 86% of total DCN), with the AIN/EN and FN contributing 6% and 7%, respectively, and the PIN/GN comprising only 1% (Fig. 3). Notably, DN volume in the ICBM152 MNI template (1,185 mm^3^) exceeded that in four individual T2-weighted human cases (972.8–1,036.3 mm^3^; mean 994.6 mm^3^), representing an approximate 19% increase likely due to template averaging effects that exaggerate small, high-contrast structures. Together, these findings reveal a progressive, species-dependent reorganization of DCN composition, characterized by disproportionate expansion of the DN across primates, with the most pronounced enlargement observed in humans.

Cross-species comparisons reveal a systematic evolutionary trend: DN relative volume increases from 22% in marmosets to 45% in macaques and 86% in humans, whereas the proportional contributions of the interposed nuclei (AIN/EN and PIN/GN) and the FN decline, with the PIN/GN showing the most pronounced reduction (22% → 16% → 1%). These findings highlight a striking, evolutionarily conserved reorganization of DCN subregions, emphasizing the human-specific expansion of the dentate nucleus, consistent with its enhanced role in cerebellar output to higher-order cortical networks.

### Species-dependent scaling of deep cerebellar nuclei relative to total cerebellar size

For total DCN volume, we used values derived from in vivo population-based datasets in each species (Marmoset: 14.1 mm^3^; Macaque: 196.3 mm^3^; Human: 1,384 mm^3^; Fig. 3, top panel; gray-highlighted) to ensure consistency with total cerebellar volume estimates (Table 1). This corresponds to an approximate 13.9-fold increase from marmoset to macaque and a further ~7.0-fold increase from macaque to human, yielding an overall ~98-fold expansion. Individual DCN subcomponents were quantified as averaged volumes across specimens. Together, these results indicate a nonuniform reorganization of cerebellar output structures across primates, characterized by disproportionate expansion of specific subcomponents, particularly the dentate nucleus in humans.

**Fig. 4 |:**
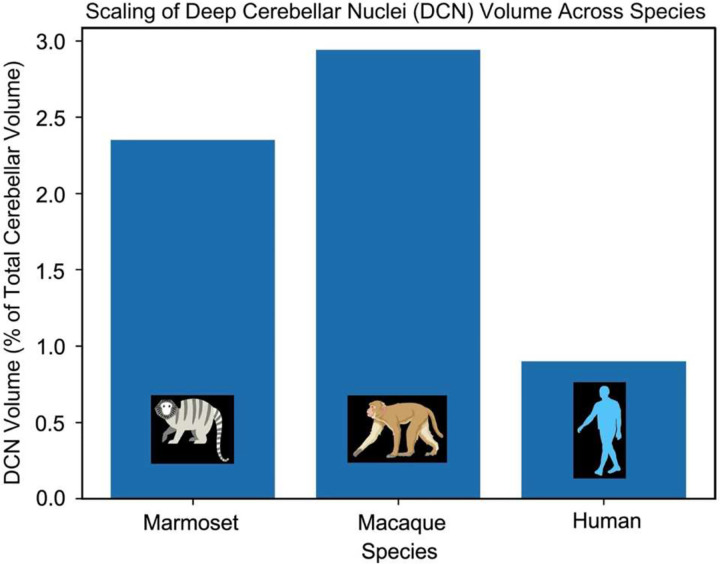
Sublinear scaling of deep cerebellar nuclei relative to total cerebellar volume Deep cerebellar nuclei (DCN) volume expressed as a fraction of total cerebellar volume across primates. Despite large increases in absolute DCN volume, their proportional contribution decreases with brain size. DCN comprise ~2.35% of total cerebellar volume in marmosets, ~2.94% in macaques, and ~0.90% in humans, indicating sublinear scaling of cerebellar output structures relative to overall cerebellar expansion.

When normalized to total cerebellar volumes derived from the same population-based datasets (Table 1; Marmoset: 599.14 mm^3^; Macaque: 6,685.42 mm^3^; Human: 154,161.1 mm^3^), distinct species-dependent scaling patterns emerge (Fig. 4). DCN comprise ~2.35% of total cerebellar volume in marmosets, ~2.94% in macaques, and ~0.90% in humans, indicating that, despite substantial absolute expansion, DCN volume does not scale proportionally with cerebellar size in humans. These findings further support a nonuniform scaling of cerebellar output structures, consistent with the pronounced dominance of the dentate nucleus in humans.

### Cross-species multimodal cerebellar atlases enable lobular comparison across primates

**Fig. 5 |:**
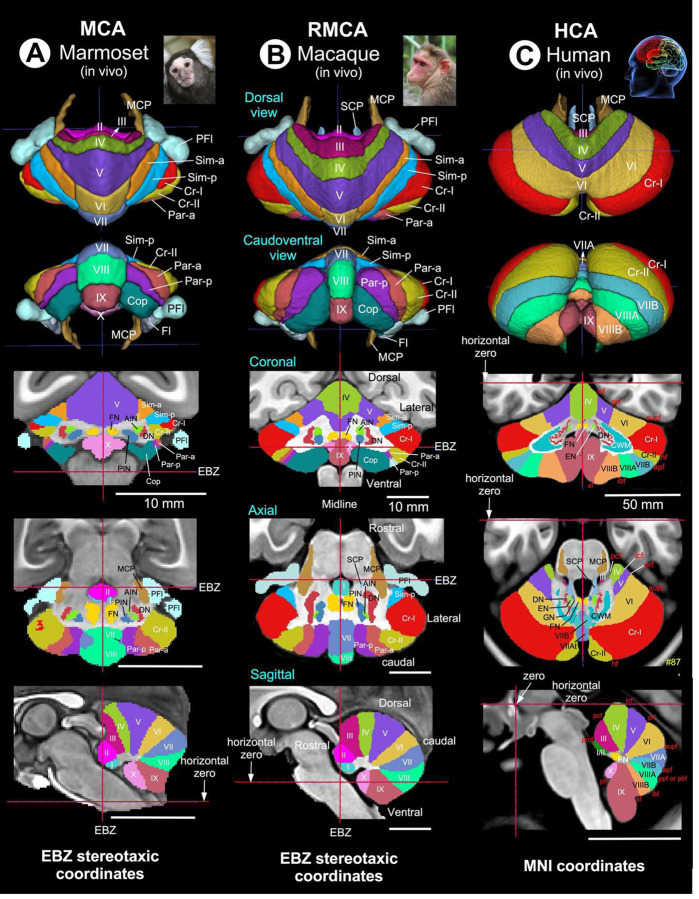
Cross-species multimodal cerebellar atlases enable lobular comparison across primates High-resolution multimodal cerebellar atlases were constructed for marmoset, macaque, and human by integrating structural MRI with histological information (see Materials and Methods). In marmoset and macaque, ex vivo segmentations of cerebellar lobules, deep cerebellar nuclei, and cerebellar peduncles were transformed into species-specific stereotaxic space and aligned to population-based in vivo templates, generating the Marmoset Cerebellar Atlas (MCA) and Rhesus Macaque Cerebellar Atlas (RMCA) (**A, B**). In humans, cerebellar segmentation was performed in MNI space (ICBM-152 template) using cytoarchitectonic priors derived from the BigBrain histological dataset registered to the same reference space, yielding the Human Cerebellar Atlas (HCA) (**C**). Together, these atlases enable consistent delineation of vermal and hemispheric lobules as well as deep cerebellar nuclei across species using standardized Larsell and Schmahmann nomenclature^23–25^. Visualization across coronal, axial, and sagittal planes supports direct anatomical comparison and provides a unified framework for cross-species volumetric analyses. ***Abbreviations:*** I-X: cerebellar lobules; AIN: anterior interposed nucleus; Cop: copula of pyramis; Cr-1: crus I (ansiform lobule); Cr-II: crus II (ansiform lobule); CWM: cerebellar white matter; DN: dentate nucleus; EN: emboliform nucleus; Fl: Flocculus; FN: fastigial nucleus; GN: globose nucleus; MCP: middle cerebellar peduncle; Par-a: paramedian lobule, anterior part; Par-p: paramedian lobule, posterior part; PFl: paraflocculus; PIN: posterior interposed nucleus; SCP: superior cerebellar peduncle; Sim-a: simple lobule, anterior part; Sim-p: simple lobule, posterior part. For the abbreviations of the fissures (red text) separating the cerebellar lobules in the human (**C**), see Supplementary Fig. 6. Scale bars: 10 mm, applies to all scale bars in A-B; 50 mm, applies to C.

We constructed multimodal, high-resolution cerebellar atlases for marmoset, macaque, and human by integrating MRI with histological information (see Methods). In marmoset and macaque, ex vivo segmentations of cerebellar lobules, deep cerebellar nuclei, and cerebellar peduncles were transformed into species-specific stereotaxic spaces and aligned to in vivo population templates, forming the Marmoset Cerebellar Atlas (MCA) and Rhesus Macaque Cerebellar Atlas (RMCA) (Fig. 5A, B). In humans, cerebellar parcellation was performed in MNI space using the ICBM-152 template, guided by surface fissures and cytoarchitectonic reconstructions from the BigBrain dataset^20^, generating the Human Cerebellar Atlas (HCA) (Fig. 5C). A detailed HCA with complete fissural and lobular delineation is provided in Supplementary Fig. 6. These atlases provide a unified framework for defining vermal and hemispheric lobules as well as deep cerebellar nuclei across primates using standardized Larsell and Schmahmann nomenclature^23–25^. Visualization in coronal, axial, and sagittal planes enables direct cross-species comparison of cerebellar architecture in a common stereotaxic reference space.

Vermal lobules (I-X) are largely conserved across species and serve as stable anatomical anchors. In contrast, hemispheric territories of the posterior lobe (lobules VI-IX) show progressive expansion and increasing lateral differentiation from marmoset to macaque to human (Fig. 5A–C; Table 2). Within this framework, Crus I and Crus II emerge as increasingly prominent lateral posterior structures, particularly in humans, where they form expanded hemispheric domains distinctly segregated from vermal cortex. Across marmoset and macaque atlases, posterior hemispheric cortex is further subdivided into species-specific territories (Simplex, Paramedian, and Copula of pyramis)^21,22^, reflecting graded hemispheric specialization that is not explicitly parcellated in the human atlas but maps topologically onto lobules VI-IX (Fig. 5A–C; Table 2). Together, these atlases establish a consistent anatomical platform for cross-species comparison of cerebellar lobular organization and provide the structural basis for subsequent volumetric and scaling analyses.

### Posterior cerebellar territories and Crus I/II collectively dominate cerebellar volume in humans

**Fig. 6 |:**
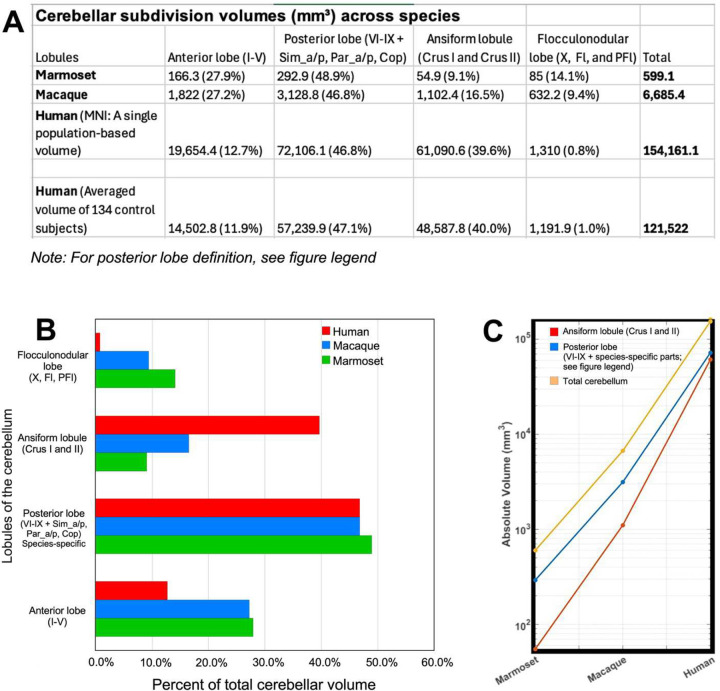
Disproportionate expansion of posterior cerebellar territories and the ansiform lobule (Crus I/II) across primates **(A)** Cerebellar subdivision volumes in marmoset, macaque, and human, shown as absolute volumes (mm^3^) and proportions of total cerebellar volume. Major subdivisions include the anterior lobe (lobules I–V), posterior cerebellar cortex, ansiform lobule (Crus I/II), and flocculonodular lobe. Posterior lobe definitions follow species-specific parcellations: in marmoset and macaque, lobules VI-IX together with simplex (Sim), paramedian (Par), and copula of the pyramis (Cop); in humans, lobules VI-IX only. Crus I/II is shown separately to highlight lateral posterior hemispheric territories. **(B)** Relative distribution of total cerebellar volume across species. The posterior cerebellar cortex constitutes a major component in all species, whereas Crus I/II accounts for a substantially larger proportion in humans (~40%) than in macaque and marmoset. **(C)** Log-scaled absolute volumes of cerebellar subdivisions across species. All subdivisions increase with brain size; Crus I/II shows the steepest scaling trajectory, exceeding total cerebellar growth and other subdivisions.

To determine whether hemispheric reorganization is accompanied by volumetric redistribution, we quantified cerebellar lobular scaling across marmosets, macaques, and humans using atlas-derived measurements (Fig. 6A–C). Lobules were grouped into major anatomical subdivisions to enable cross-species comparison, with Crus I/II analyzed separately as a lateral extension of the posterior hemispheric cortex.

Across species, posterior cerebellar territories constituted the largest proportion of cerebellar volume, although their anatomical definitions differ between species (Fig. 6A, B). In marmosets and macaques, the posterior cerebellar cortex (lobules VI-IX, including Sim, Par, and Cop) accounted for 48.9% and 46.8% of total cerebellar volume, respectively. In humans, where these species-specific subdivisions are not defined, lobules VI-IX similarly comprised 46.8% of cerebellar volume. Notably, this was closely matched by a marked expansion of Crus I/II, which reached 39.6% and approached the posterior lobe in relative contribution, forming a co-dominant hemispheric territory. In contrast, anterior lobules (I-V) showed a progressive reduction in relative volume (27.9% in marmosets, 27.2% in macaques, and 12.7% in humans), while the flocculonodular lobe decreased substantially (14.1%, 9.4%, and 0.8%, respectively). Together, these results indicate a progressive redistribution of cerebellar volume toward posterior hemispheric territories, with the most pronounced relative expansion in Crus I/II.

We next examined absolute scaling on a logarithmic scale (Fig. 6C). Because posterior lobe definitions differ across species, encompassing lobules VI-IX with inclusion of Sim, Par, and Cop in marmosets and macaques but restricted to lobules VI-IX in humans, these measurements reflect species-specific parcellations and should be interpreted as comparative scaling trends rather than strictly homologous volumes. Within this framework, posterior cerebellar territories increased approximately 10.7-fold from marmosets to macaques and ~23-fold from macaques to humans (~246-fold overall). Crus I/II exhibited a markedly steeper trajectory, expanding ~20-fold and ~55-fold across the same transitions (>1,100-fold overall), exceeding the scaling of total cerebellar volume (~11-fold and ~23-fold). Thus, despite differences in anatomical definitions, Crus I/II display the steepest absolute scaling among cerebellar subdivisions, highlighting a disproportionate expansion of lateral posterior hemispheric cortex in the human cerebellum.

### Relative Scaling of Cerebellar Lobules Is Preserved Across Native and Template MRI

To validate population-level cerebellar compartmentalization, the Human Cerebellar Atlas (HCA), derived from a population-based MNI template, was applied to in vivo T1-weighted MRI from 134 healthy individuals spanning a range of ages and both sexes (Supplementary Table 1). This enabled lobular parcellation in native space and estimation of average volumetric distributions across subjects. Across the cohort, cerebellar subdivisions closely matched the template-derived proportions (Fig. 6A). The posterior cerebellum (lobules VI-IX, excluding Crus I/II) and Crus I/II together accounted for 87.1% of total cerebellar volume. Specifically, the anterior lobe (lobules I-V) comprised 11.9%, posterior lobules 47.1%, Crus I/II 40.0%, and the flocculonodular lobe 1.0%. These values closely matched MNI template estimates (12.7%, 46.8%, 39.6%, and 0.8%, respectively), indicating strong preservation of relative cerebellar scaling across datasets. Although absolute volumes were higher in MNI space than in cohort-based estimates, consistent with known template-related inflation effects^26,27^, proportional relationships remained stable across individuals. This suggests that cerebellar compartmental organization is robust to demographic variation and imaging space, supporting the reliability of atlas-based scaling analyses.

## Discussion

The cerebellum contributes to distributed sensorimotor, cognitive, and affective processing through closed-loop cortico-cerebellar circuits, in which the cerebellar cortex provides input processing and the deep cerebellar nuclei (DCN) constitute the sole output channels. Using multimodal, histology-validated cross-species atlases integrating MRI and iron-sensitive imaging, we show that these input and output compartments scale nonuniformly across marmoset, macaque, and human. Specifically, cerebellar cortical territories expand disproportionately relative to DCN, revealing asymmetric scaling of cerebellar architecture across primates. Posterior hemispheric regions, particularly lobules VI-IX and Crus I/II, show preferential expansion, whereas DCN subregions exhibit selective rather than proportional enlargement. Together, these findings identify a systems-level organizational principle in which cerebellar input and output pathways scale independently, reshaping integration within distributed brain networks.

DCN MRI contrast closely reflects underlying iron content across species. T2 hypointensity in macaque and human DCN, contrasted with reduced or inverted signal in marmosets, corresponds to Perls’ iron staining and is consistent with prior work linking susceptibility effects to tissue iron^28–30^. These results support iron-sensitive MRI as a biologically meaningful marker of DCN microstructure. Given iron’s role in oxidative metabolism and mitochondrial function, species differences in iron accumulation may reflect variation in metabolic demand within cerebellar output nuclei. These effects are important for interpreting DCN imaging across species and for translational studies of cerebellar pathology. Quantitative susceptibility mapping may further refine the relationship between MRI contrast and cellular iron content^31^.

High-resolution, histology-validated MRI delineated the dentate (DN), interposed, and fastigial nuclei in marmosets, macaques, and humans (Fig. 2). In nonhuman primates, T2-weighted imaging, supplemented with MTR and MAP-MRI metrics, reliably distinguished nuclei, while histological markers (SMI-32, NeuN, AChE, Nissl) confirmed boundaries and highlighted microanatomical features such as cell-sparse zones and differential neuropil labeling. Marmosets exhibited a small accessory fastigial nucleus (aFN), illustrating subtle species-specific variation. Extending this approach to humans, BigBrain-informed segmentation enabled clear identification of all major DCN, including the highly folded dentate nucleus, in postmortem datasets^20^. Together, these results demonstrate that histology-guided MRI provides a robust framework for bridging nonhuman primate and human cerebellar mapping, enabling precise cross-species comparisons of structure, connectivity, and function^3,32,33^.

Quantitative volumetric analyses reveal selective enlargement of the dentate nucleus (DN) across species, with humans showing the most pronounced dominance. In marmosets, DCN volumes are relatively balanced, whereas macaques and humans exhibit disproportionate DN expansion, reflecting the DN’s central role as the principal output of the lateral cerebellum and its extensive connectivity with higher-order cortical systems^3,13,32^. Our marmoset findings differ from those reported by Zhu and colleagues^34^, who described the DN as the largest DCN subregion, exceeding the combined volume of the interposed and fastigial nuclei. In their study, the DN was delineated together with the anterior interposed (emboliform) nucleus, likely contributing to larger estimated DN volumes and DN-to-other-nuclei ratios. By contrast, our high-resolution MRI guided by histological ground truth enabled precise delineation of individual DCN subregions (Fig. 2), indicating that pronounced DN enlargement is not a defining feature of the marmoset but emerges progressively in larger-brained primates.

In humans, DN specialization is most pronounced, accounting for 1032.7 mm^3^ (86% of total DCN volume), whereas the fastigial and interposed nuclei contribute only minor proportions (Fig. 3). This pattern aligns with postmortem volumetric studies reporting DN contributions of ~77%^35^ and histology-based cytoarchitectonic analyses demonstrating a predominance of the DN over other nuclei^36^. High-resolution imaging studies further corroborate this DN dominance^37,38^, although absolute DN volumes vary due to methodological differences, including partial-volume effects and limited resolution^39^. Despite these differences, the relative dominance of the DN emerges as a robust feature of human cerebellar organization. Transcriptomic analyses indicate selective amplification of excitatory projection neurons within the DN, providing a cellular mechanism supporting increased output capacity^40,41^.

Beyond volumetric dominance, dentate nucleus enlargement has direct implications for cerebellar circuit architecture. As the principal output of the lateral cerebellum, the DN participates in closed-loop circuits linking cerebellar processing with distributed association cortices, including prefrontal and posterior parietal regions, through thalamic relays^6,13–16^. Its internal organization into motor and nonmotor territories, featuring differentiated projection patterns to higher-order cortical regions^13^, suggests that selective DN expansion increases the capacity and diversity of cerebellar output, supporting predictive and integrative computations extending beyond motor control^3,42^. In contrast, the interposed and fastigial nuclei, which are primarily associated with sensorimotor and axial functions^43–45^, with the exception of the posterior interposed nucleus, scale more modestly across species. This divergence reinforces the view that cerebellar evolution preferentially amplifies lateral hemispheric-dentate circuits interfacing with association cortical networks rather than uniformly expanding all output channels.

Cerebellar cortical expansion mirrors, but exceeds, deep nuclear scaling, with the strongest effects localized to posterior hemispheric territories. Across primates, lobules VI-IX and Crus I/II show progressive expansion and increasing hemispheric differentiation. In marmosets, the posterior cortex remains relatively compartmentalized with limited hemispheric elaboration, whereas in macaques, hemispheric territories become more clearly segregated, with Crus I/II forming distinct lateral domains. In humans, the posterior cerebellar cortex emerges as a dominant, continuous hemispheric structure, with Crus I/II representing major lateral expansions largely dissociated from vermal organization. These anatomical changes align with cerebro-cerebellar circuits linking posterior cerebellum to association cortices via pontine and thalamic pathways^5,32,46^. Functional imaging studies consistently implicate these regions, particularly Crus I/II, in executive, attentional, and social-cognitive processes^12^. Together, these findings indicate that cerebellar cortical expansion preferentially enhances integrative and associative processing capacity, rather than uniformly scaling cerebellar output.

Nonuniform scaling of cerebellar compartments reflects functional specialization across primates. Vermal and interposed-fastigial circuits are primarily associated with posture, locomotion, and visuomotor control, supporting core sensorimotor demands across species^43,45^. In contrast, posterior hemispheric-dentate circuits show disproportionate expansion and increased coupling with association cortices. Phylogenetic evidence further supports coordinated expansion of lateral cerebellum and association cortex in anthropoid primates^9,47^. These circuits have been implicated in predictive processing, sequencing, and adaptive behavioral control^3,32^, functions that may be particularly relevant in complex ecological and social environments^48^. Notably, the disproportionate expansion of posterior cerebellar cortex relative to the DCN suggests a shift toward enhanced cortical processing capacity without equivalent scaling of nuclear output structures. Together, these findings support a model of mosaic cerebellar evolution in which cortical and nuclear compartments exhibit differential scaling while remaining functionally integrated within distributed brain systems.

Despite the novel insights provided by our cross-species cerebellar atlases, several limitations should be acknowledged. First, the study is based on a modest number of specimens per species (n = 5), which may limit the ability to fully capture inter-individual variability, including potential sex- or age-related differences. Nevertheless, the combination of high-resolution MRI and histological delineation for each case ensures detailed and reliable anatomical measurements, supporting the consistency of the observed cross-species patterns. Second, subtle differences in tissue processing, scan resolution, or segmentation protocols across species could introduce minor measurement variability. Third, our focus on volumetric and lobular comparisons does not directly assess microstructural, connectivity, or functional differences, which are essential for understanding the full scope of cortico-cerebellar integration. Finally, although the study highlights broad patterns of posterior expansion and altered cortical-nuclear scaling, evolutionary interpretations remain correlational; additional developmental, functional, and behavioral data will be needed to link structural changes to cognitive or sensorimotor adaptations. These limitations underscore the need for future studies integrating larger sample sizes, multimodal functional imaging, and finer-scale histological analyses to refine our understanding of cerebellar evolution in primates.

## Conclusion

Using multimodal MRI-histology atlases spanning marmoset, macaque, and human, we show that cerebellar expansion is characterized by nonuniform scaling of input-output architecture rather than homogeneous enlargement. Posterior cortical territories expand disproportionately relative to anterior lobules and the deep cerebellar nuclei, accompanied by progressive lateral consolidation of hemispheric domains. Subdivisions associated with lobule VII, particularly Crus I and II, undergo marked hemispheric elaboration, whereas anterior vermal-hemispheric organization remains comparatively conserved. Importantly, cortical enlargement is not matched by proportional expansion of the deep cerebellar nuclei, revealing a shift in the balance between cortical input and nuclear output. These multimodal atlases enable precise cross-species correspondence between imaging features and underlying cellular architecture. Evolutionarily, cerebellar enlargement in primates reflects selective architectural remodeling rather than uniform growth. Preferential expansion of posterior hemispheric cortex and dentate output pathways parallels the elaboration of higher-order cortical systems, indicating coordinated reorganization of cerebro-cerebellar circuits. Rather than scaling passively, the cerebellum emerges as a selectively reorganized structure supporting increasingly complex integrative processing.

## Materials and Methods

### Non-human primate Specimens and Ethical Compliance

Brains from two adult common marmosets (Callithrix jacchus, male 340 g; female 283 g) and one adult rhesus macaque (Macaca mulatta, male 13.55 kg) were obtained from the National Institute of Mental Health (NIMH/NIH) for our MRI and histological analyses. These specimens were already perfusion fixed as part of prior studies at NIMH; we did not perform any live experiments or perfusions on these animals for our research. The marmosets had previously been used in transgenic studies, and the macaque in behavioral studies. Animals had been anesthetized with sodium pentobarbital and perfused transcardially with 0.5 L heparinized saline, followed by 1–2 L (marmosets) or 4 L (macaque) of 4% paraformaldehyde in 0.1 M phosphate buffer (pH 7.4). Brains were removed, photographed, post-fixed for 8–24 h, and stored in PBS with sodium azide until MRI. Specimens were rinsed in PBS before scanning to reduce residual fixative effects. All procedures conformed to the Guide for the Care and Use of Laboratory Animals (National Research Council) and were approved by the NIMH/NIH Institutional Animal Care and Use Committee.

### Ex Vivo MRI Acquisition in Marmosets

For MRI acquisition, fixed brains (cases 1–2) were positioned in 3D-printed molds (Fig. 1) within 30-mm cylindrical containers filled with Fomblin and vacuum-degassed to eliminate air bubbles. Samples were sealed and scanned on a Bruker 7T/300 mm horizontal-bore MRI system using a 30-mm quadrature Millipede coil (ExtendMR). The optimal MRI acquisition parameters differed slightly between the two cases. Detailed descriptions of marmoset and macaque sample preparation and MRI protocols have been reported previously^49,50^.

For cases 1 and 2, ex vivo MAP-MRI data were acquired at 150-μm isotropic resolution using a 3D diffusion spin-echo EPI sequence. Case 1 was acquired with a 288 × 184 × 184 imaging matrix; FOV, 4.32 × 2.76 × 2.76 cm; TE/TR = 48/650 ms; 10 segments; 1.33 partial Fourier acceleration; δ/Δ = 6/28 ms; and 256 DWIs across 11 b-value shells (100–10,000 s/mm^2^) with 7–40 uniformly distributed gradient directions per shell. Case 2 was acquired with a 256 × 160 × 160 imaging matrix; FOV, 3.84 × 2.40 × 2.40 cm; TE/TR = 43/1400 ms; 8 segments; 1.25 partial Fourier; δ/Δ = 8/20 ms; and 112 DWIs across six b-value shells (100–10,000 s/mm^2^) with 3–36 directions per shell; two averages were acquired at b = 10,000 s/mm^2^ to improve SNR. In both cases, diffusion directions were uniformly sampled on the unit sphere^51,52^.

Magnetization transfer (MT)- prepared 3D gradient echo images were also acquired at 150-μm isotropic resolution (TE/TR = 3.7/37 ms; 15° flip angle) using a 2-kHz offset, 12.5-ms Gaussian saturation pulse (6.74 μT peak amplitude; 540° flip angle), with four averages for MT-on and MT-off scans. Total acquisition times were 85 h 20 min (case 1) and 74 h 40 min (case 2) for MAP-MRI, and 11 h 8 min (case 1) and 8 h 31 min (case 2) for MT imaging.

A third perfusion-fixed marmoset brain sample (Case 3, female, 289 g) was prepared for MRI in a manner similar to the other two specimens. The sample was scanned on a vertical-bore 7T Bruker MRI system using a 30 mm millipede RF coil and a 3D diffusion spin-echo EPI sequence. Data were acquired with 86 μm isotropic resolution.

### Ex Vivo MRI Acquisition in the Macaque

A 3D structural MRI of the fixed ex vivo macaque brain was first obtained and reoriented to the stereotaxic plane of the D99 atlas^49,53^. The aligned volume was used to 3D-print a custom cylindrical brain mold for stable positioning during scanning. The specimen was placed in a 70-mm cylindrical container filled with Fomblin, vacuum-degassed for 4 h to remove air bubbles, sealed, and scanned on a Bruker 7T/300 mm horizontal-bore MRI system using a 72-mm quadrature RF coil. All MRI volumes were registered to the D99 atlas using affine and nonlinear transformations.

MAP-MRI data were acquired at 200-μm isotropic resolution (375 × 320 × 230 matrix; FOV, 7.5 × 6.4 × 4.6 cm; TE/TR = 50/650 ms; 8 segments; 1.33 partial Fourier acceleration; δ/Δ = 6/28 ms) with 112 DWIs across six b-value shells (100–10,000 s/mm^2^) and 3–36 uniformly distributed directions per shell. MT-prepared 3D gradient echo images were acquired at 250-μm isotropic resolution (TE/TR = 3.5/37 ms; 15° flip angle) using a 2-kHz offset, 12.5-ms Gaussian saturation pulse (6.74 μT peak amplitude; 540° flip angle), with two averages for MT-on and MT-off scans. Total acquisition times were 93 h 20 min (MAP-MRI) and 6 h 18 min (MT).

### Diffusion modeling and microstructural metrics in marmoset and macaque

Post-processing of MRI data was identical across species. Diffusion-weighted images were processed using the TORTOISE software package^54^ to correct for Gibbs ringing, signal drift, and distortions arising from magnetic field inhomogeneity and eddy currents, with the MTR image serving as the structural reference. The mean apparent propagator was estimated voxelwise using a fourth-order MAP series approximation implemented in MATLAB. From the diffusion tensor model, we derived fractional anisotropy (FA), mean diffusivity (MD), axial diffusivity (AD), radial diffusivity (RD), and tensor shape coefficients (CL, CP, CS). We derived MAP-MRI metrics, including propagator anisotropy (PA), return-to-origin (RTOP), return-to-axis (RTAP), and return-to-plane (RTPP) probabilities, non-Gaussianity (NG), and the non-diffusion-weighted amplitude image (T2-weighted contrast). Orientation distribution functions (ODFs) and fiber ODFs were estimated and visualized using MRtrix3^55^. MTR maps were computed from MT-on and MT-off images and provided high gray-white matter contrast for reliable registration to diffusion volumes.

### Histology and immunohistochemistry

**Fig. 7 |:**
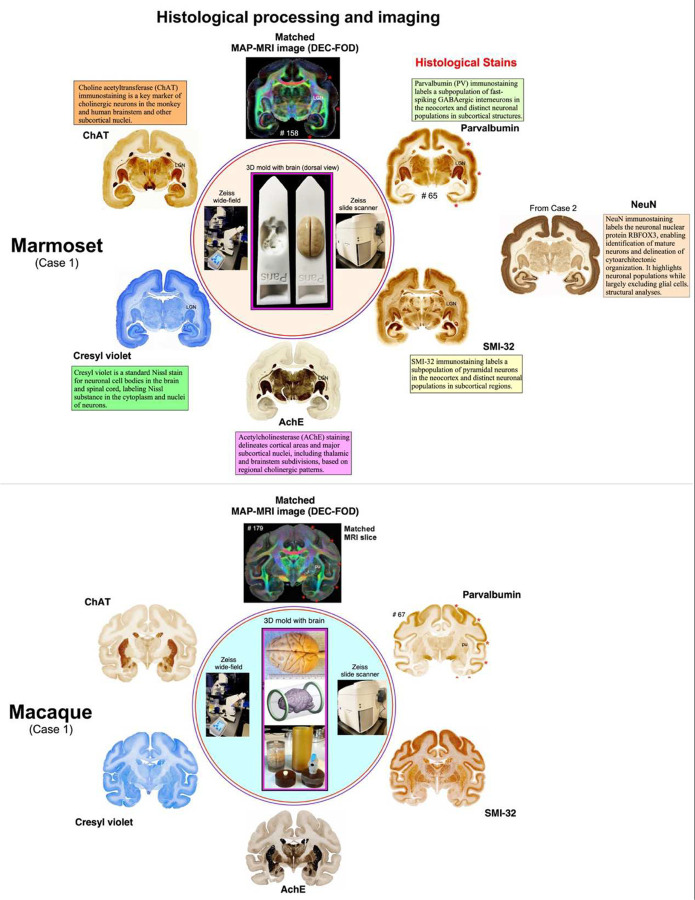
Complementary histological staining and MRI correspondence in marmoset and macaque Complementary histological staining and MRI datasets were used to establish cross-modal anatomical correspondence across primate cerebellar and cortical structures. In marmoset (Case 1), serial coronal frozen sections (50 μm) spanning the rostrocaudal axis of the brain were collected and assigned to interleaved staining series to preserve spatial continuity across modalities. Representative sections at the level of the anterior temporal cortex include Nissl staining, acetylcholinesterase (AChE) histochemistry, and immunohistochemistry for parvalbumin (PV), SMI-32 (non-phosphorylated neurofilament H), and choline acetyltransferase (ChAT). In marmoset (Case 2) and macaque, additional NeuN immunostaining and Perls’ Prussian blue histochemistry were performed for cytoarchitectonic and iron visualization, respectively. High-resolution brightfield images were acquired using a Zeiss Axioscan Z1 slide scanner and wide-field microscopy. Histological sections were manually matched to corresponding MAP-MRI and structural MRI datasets from the same specimens using conserved anatomical landmarks, including sulcal fundi, gyral crowns, ventricular boundaries, and deep gray matter structures. This cross-modal registration reveals strong correspondence between MRI signal and histological architecture across cortical and subcortical regions. Anatomical landmarks such as sulcal and gyral patterns (red stars) and ventricular contours are consistently aligned across modalities, enabling reliable identification of deep structures, including cerebellar nuclei, within a unified spatial framework. In addition, specimen-specific 3D brain molds were used during ex vivo imaging to preserve native brain geometry and support accurate MRI–histology alignment.

Following MRI acquisition, brains were cryoprotected, sectioned, and processed for histological staining. Histological procedures were identical for marmoset and macaque specimens and are described in detail elsewhere^49,50^ (Fig. 7). Briefly, serial sections were processed for Nissl staining, acetylcholinesterase (AChE) histochemistry, and immunohistochemistry using antibodies against parvalbumin (PV), SMI-32 (nonphosphorylated neurofilament H), and choline acetyltransferase (ChAT). In the marmoset case 2 and the macaque, an additional staining for NeuN and Perls’ Prussian blue (iron) was performed. Immunoreactions were visualized using the avidin–biotin complex method with diaminobenzidine as the chromogen. Sections were mounted, dehydrated, cleared, and coverslipped using standard procedures. Detailed protocols and antibody information are described previously^49,50^, and the software used in this study is summarized in Table 3.

### Data analysis

High-resolution images of all stained sections were acquired using a Zeiss wide-field microscope and an Axioscan Z1 slide scanner (5× objective) (Fig. 7). Digital images were adjusted for brightness and contrast and manually aligned to the corresponding diffusion-derived maps (DTI and MAP-MRI metrics), estimated T2-weighted (non-diffusion-weighted) images, and MTR volumes to facilitate visualization and 3D segmentation of cerebellar lobules, deep cerebellar nuclei, and the cerebellar peduncles (Figs. 2, 5; Supplementary Fig. 1). Accurate alignment was achieved without resampling the MRI volumes. Precise orientation of the intact brain specimen relative to sagittal MRI images was performed prior to sectioning, enabling consistent matching of sulci, gyri, and deep ROIs across MRI and histological sections (Fig. 7)^49,50^.

### Cerebellar segmentation, 3D atlas generation, and volumetric analysis

In marmoset and macaque, deep cerebellar nuclei (dentate nucleus [DN], anterior interposed nucleus [AIN], posterior interposed nucleus [PIN], and fastigial nucleus [FN]) and vermal and hemispheric subdivisions of cerebellar lobules were manually segmented from ex vivo coronal MRI volumes (primarily T2-weighted and complementary MAP-MRI contrasts) using ITK-SNAP^56^. MRI-defined boundaries were validated against matched high-resolution histological sections (SMI-32, NeuN, AChE, and Nissl stains) and informed by previously described cerebellar anatomical frameworks in these species, with nomenclature aligned to prior studies^21,22,24^. Segmented regions were reconstructed in three dimensions and visualized using ITK-SNAP^56^ and SUMA^57^ to define spatial relationships among cerebellar cortical and nuclear subdivisions. These reconstructions formed the basis of the Marmoset Cerebellar Atlas (MCA) and Rhesus Macaque Cerebellar Atlas (RMCA). Ex vivo MCA and RMCA segmentations were subsequently transformed into species-specific stereotaxic space and aligned to population-based in vivo templates for atlas generation (Fig. 5A, B). All segmentations were manually reviewed and refined to ensure accurate areal extent and architectonic boundaries of cerebellar lobules and deep nuclei, with particular reference to fissural landmarks and histological features. Spatial normalization and segmentation were performed in AFNI^57,58^ using pipelines optimized for high-resolution ex vivo imaging. The resulting population-based in vivo templates constitute the standard MCA and RMCA (Fig. 5A, B) used for quantitative analyses and will be made publicly available through the AFNI platform (link forthcoming).

For humans, cerebellar lobules and deep cerebellar nuclei (DN; emboliform nucleus [EN, corresponding to AIN]; globose nucleus [GN, corresponding to PIN]; and FN) were segmented with reference to established anatomical descriptions^23,36^. The T1-weighted MNI_icbm152 nonlinear template^59,60^ served as the stereotaxic reference space. Because deep nuclei are not well resolved in standard T1-weighted MNI templates, high-resolution cytoarchitectonic data from the BigBrain dataset^20^ were co-registered to MNI space to refine nuclear boundaries (Figs. 2C, F; Supplementary Figs. 4, 7). In addition, in vivo diffusion data, including direction-encoded color (DEC) volumes from the Human Connectome 1.0 Project^61^, were nonlinearly registered to this space (Supplementary Fig. 7B) to aid delineation of cerebellar peduncles and intracerebellar fiber orientations (analyses will be reported in a separate manuscript). Integration of these multimodal datasets enabled the generation of the Human Cerebellar Atlas (HCA) (Fig. 5C, Supplementary Fig. 6).

To generate a flatmap representation of the human cerebellum, we first aligned the MNI 2009c cerebellar volume to the SUIT cerebellar template^62^ using nonlinear warping implemented in 3dQwarp (AFNI). The resulting deformation field was then applied to the HCA (Human Cerebellar Atlas) with nearest neighbor interpolation to bring it into the SUIT volumetric space. Next, the transformed atlas was projected onto the SUIT surface representation using the vol2surf plugin within the AFNI GUI, sampling voxel values along the cortical ribbon between the SUIT white matter and pial surfaces with a modal (most frequent value) mapping approach. Finally, the surface-projected atlas was smoothed on the pial surface using 10 mm modal smoothing with SurfLocalstat to reduce local discontinuities while preserving discrete parcellation boundaries (Supplementary Fig. 6).

Each atlas includes detailed segmentation of vermal and hemispheric lobules and deep cerebellar nuclei (DCN), standardized according to Paxinos/Larsell/Schmahmann nomenclature^21–25^, and provides multiplanar visualizations in coronal, axial, and sagittal orientations in stereotaxic or MNI coordinates (Fig. 5A–C). For the HCA, detailed fissural landmarks separating lobules are also provided (Supplementary Fig. 6). Homologous lobules and nuclei were identified across marmoset, macaque, and human templates based on structural landmarks and histological validation, enabling reliable cross-species comparisons (Table 2). The 3D cerebellar atlas templates in these species, and the script for atlas registration of in vivo scans, are now available for download through the AFNI and SUMA websites at https://afni.nimh.nih.gov/…………………. (link forthcoming). The AFNI software can install this simply with the @Install_SAM_Marmoset command.

For consistency, volumetric measurements of cerebellar lobules and deep cerebellar nuclei were obtained from finalized 3D atlas segmentations derived from species-specific, population-based templates (Figs. 5, Supplementary Fig. 6; Table 1; Figs. 3, 4, 6). This approach provides a standardized framework for cross-species comparison while minimizing biases arising from individual variability. The resulting digital atlases define stereotaxic reference volumes and surface parcellations suitable for comparative cerebellar mapping and integration into neuroimaging pipelines.

### Additional Cases for Cross-Species Quantitative DCN Analysis

To enhance cross-species volumetric comparisons of the deep cerebellar nuclei (DCN), we supplemented our primary datasets with independently acquired cases from marmosets, macaques, and humans, yielding five cases per species (Fig. 3, top panel). In the marmoset, beyond the three ex vivo T2-weighted MRI datasets generated in this study (Cases 1–3), we incorporated the population-averaged in vivo MBM_v3.0 T2-weighted template (200 μm resolution)^63^ and a high-resolution ex vivo MBM_v5.0 dataset (80 μm resolution)^34^ (Supplementary Fig. 5).

In the macaque (Fig. 3, top panel), we combined our ex vivo T2-weighted dataset (200 μm resolution; Case 1) with an ex vivo T2-weighted (G12) dataset registered to the NMT v2.0 population-based template^64^, and three quantitative susceptibility mapping (QSM) datasets (400 μm resolution) provided by Atsushi Yoshida^31^. QSM contrast improved visualization and delineation of DCN subregions. In both marmosets and macaques, DCN boundaries were segmented serially on T2-weighted and QSM sections with direct reference to closely matched histological sections obtained from our own ex vivo cases (SMI-32, NeuN, and Nissl staining), ensuring species-specific anatomical fidelity.

For humans, in addition to the MNI T1-weighted template, we analyzed four independent T2-weighted MRI volumes (800 μm isotropic resolution) spanning different ages and both sexes (Fig. 3, top panel), obtained from the OpenNeuro (Chai et al., 2025). DCN segmentation was performed on serial T2-weighted sections using the BigBrain template registered to subject space as a histological reference^20^ (Supplementary Fig. 5). Together, this multi-source, multimodal framework enabled robust evaluation of DCN volumetry across species while minimizing template- and cohort-specific bias.

## Supplementary Material

1

## Figures and Tables

**Table 1.** Lobule-specific cerebellar volumes in marmoset, macaque, and human.

Volume of the cerebellum in the Marmoset, Macaque, and Human in mm3																					
Lobules	I	II	III	IV	V	VI	VII	VIII	IX	X	Crus I	Crus II	Sim_a	Sim_p	Par_a	Par_p	Cop	Fl	PFl	Total	DCN
**Marmoset**	1.94	17.6	31.1	35.7	80	51.5	42.6	26.4	31.1	16.6	9.3	45.6	18	29.1	27.5	25.6	41.1	8.2	60.2	**599.14**	**14.1**
																					
**Macaque**	32.9	135.1	390.2	416.1	847.7	285.3	214.7	180.1	219.9	76.4	616.1	486.3	282.9	395.9	378.4	436.4	735.2	77	478.8	**6685.42**	**196.3**
																					
**Human**	161		1903.8	7791.6	9798	22330	15246	25230.6	9299.5	1310	38310.4	22780.2								**154161.1**	**1384**

Absolute volumes (mm^3^) are shown for individual cerebellar lobules and hemispheric subdivisions derived from species-specific atlases (Fig. 5A–C). Lobules I-X follow conventional numbering, with Crus I and Crus II (ansiform lobule) listed separately. In marmoset and macaque, additional hemispheric subdivisions (simplex, paramedian, copula of the pyramis, flocculus, and paraflocculus) are defined according to nonhuman primate anatomical conventions^21,22^. In humans, consistent with Schmahmann and colleagues^23^, the flocculonodular lobe comprises lobule X (nodulus) and the flocculus; in our human cerebellar atlas (HCA), both the nodulus (vermal component) and the flocculus (hemispheric component) are incorporated within lobule X, and a distinct paraflocculus is not separately parcellated. Total cerebellar volume reflects the summed cortical subdivisions; deep cerebellar nuclei (DCN) volumes are shown separately.

**Table 2 |:** Cross-species correspondence of posterior cerebellar lobules

Lobule (Vermis)	Hemisphere	Hemisphere	Hemisphere
	MARMOSET	MACAQUE	HUMAN
I	-	-	-
II	II	II	II
III	III	III	III
IV	IV	IV	IV
V	V	V	V
VI	Simplex (Sim-a)	Simplex (Sim-a/p)	VI
VII (or VIIA)	Simplex (Sim-p), Crus I, Crus II	Simplex (Sim-p), Crus I, Crus II	Crus I
VII (or VIIB)	Paramedian (Par-a)	Paramedian (Par-p)	VIIB, Crus II
VIII (or VIIIA)	Paramedian (Par-a), Cop	Paramedian (Par-p), Cop	VIIIA
VIII (or VIIIB)	Paramedian (Par-p), Cop	Paramedian (Par-p), Cop	VIIIB
IX	Copula of pyramis (Cop)	Copula of pyramis (Cop)	IX
X(Nodulus)	Flocculus (Fl), Paraflocculus (PFl)	Flocculus (Fl), Paraflocculus (PFl)	Flocculus (or X)

Correspondence between vermal and hemispheric cerebellar lobules in marmoset, macaque, and human reveals progressive posterior hemispheric reorganization across primates (See Fig. 5A–C). Vermal lobules (I-X) are aligned using conserved cerebellar nomenclature across species. In marmosets and macaques, hemispheric posterior territories are subdivided into Simplex (Sim-a, Sim-p), Paramedian (Par-a, Par-p), and the Copula of pyramis (Cop). These regions correspond topologically to human posterior lobules VI-IX.

Sim-a and Sim-p map onto hemispheric expansions of lobule VI and anterior VII (VIIA), Par-a and Par-p correspond to lobules VII-VIII, and Cop corresponds to more caudal hemispheric territories approximating lobules VIII-IX in humans.

Crus I and Crus II represent laterally expanded hemispheric domains associated with lobule VII (Fig. 5A–C). In the macaque, these territories are spatially segregated from vermal VIIA and do not form a continuous surface extension, whereas in the marmoset they remain partially adjacent, reflecting reduced hemispheric elaboration. In humans, Crus I and Crus II form large, highly expanded hemispheric territories that are clearly separated from vermal cortex.

Thus, cross-species alignment reflects topological and developmental correspondence rather than strict one-to-one anatomical homology, highlighting progressive lateral displacement and hemispheric expansion of posterior cerebellar cortex. Importantly, differences in hemispheric parcellation schemes across species (e.g., Sim/Par/Cop in non-human primates vs. expanded Crus territories in humans) reflect atlas conventions rather than discontinuities in underlying cerebellar organization. Anatomical nomenclature follows previously published atlases and studies^21–24^.

**Table 3.** Antibodies and Computational Tools for MRI-Histology Integration, Segmentation, and Atlas Construction.

ONLINE RESOURCE (Histology and Software)	REFERENCES	IDENTIFIER (Online links)
**Histology-Immunohistochemistry (antibodies)**	Saleem et al. (2023)	
Anti-nonphosphorylated neurofilament H		https://www.biolegend.com/
(clone SMI-32, Cat # 801701		
anti-Parvalbumin antibody (Cat. # P3088)		https://www.sigmaaldrich.com/
anti-choline acetyltransferase antibody (Cat. # AB144P)		https://www.sigmaaldrich.com/
**Software**		
ITK-SNAP version 4.0	Yushkevich et al. (2006)	http://www.itksnap.org/pmwiki/pmwiki.php
Mrtrix 3.0.1	Tournier et al. (2012)	https://www.mrtrix.org/
Canvas X Draw 7.0.3	Saleem et al. (2021; 2023)	https://www.canvasgfx.com/products/canvas-x-draw
Adobe Photoshop version 24.2	Saleem et al. (2021; 2023)	https://www.adobe.com/
AFNI Version 22.1.10	Cox et al. (1996)	https://afni.nimh.nih.gov/

This table lists the primary antibodies used for immunohistochemical staining, as well as the software tools and in-house pipelines used for image processing, cerebellar region segmentation, 3D visualization, and atlas reconstruction. (e.g., ITK-SNAP for manual segmentation; and AFNI with SUMA for surface visualization and spatial normalization).
