# Supplementary material for: Asymmetric scaling of cerebellar cortex and deep nuclei reshapes input-output architecture across primates
Source: Res Sq. 2026 Apr 28:rs.3.rs-9464995. Preprint. [Version 1] doi: 10.21203/rs.3.rs-9464995/v1 (PMC13142638; doi:10.21203/rs.3.rs-9464995/v1)
Supplement: 1 [file NIHPPRS9464995V1-supplement-1.pdf]

## Supplementary Information

**Supplementary Fig. 1**

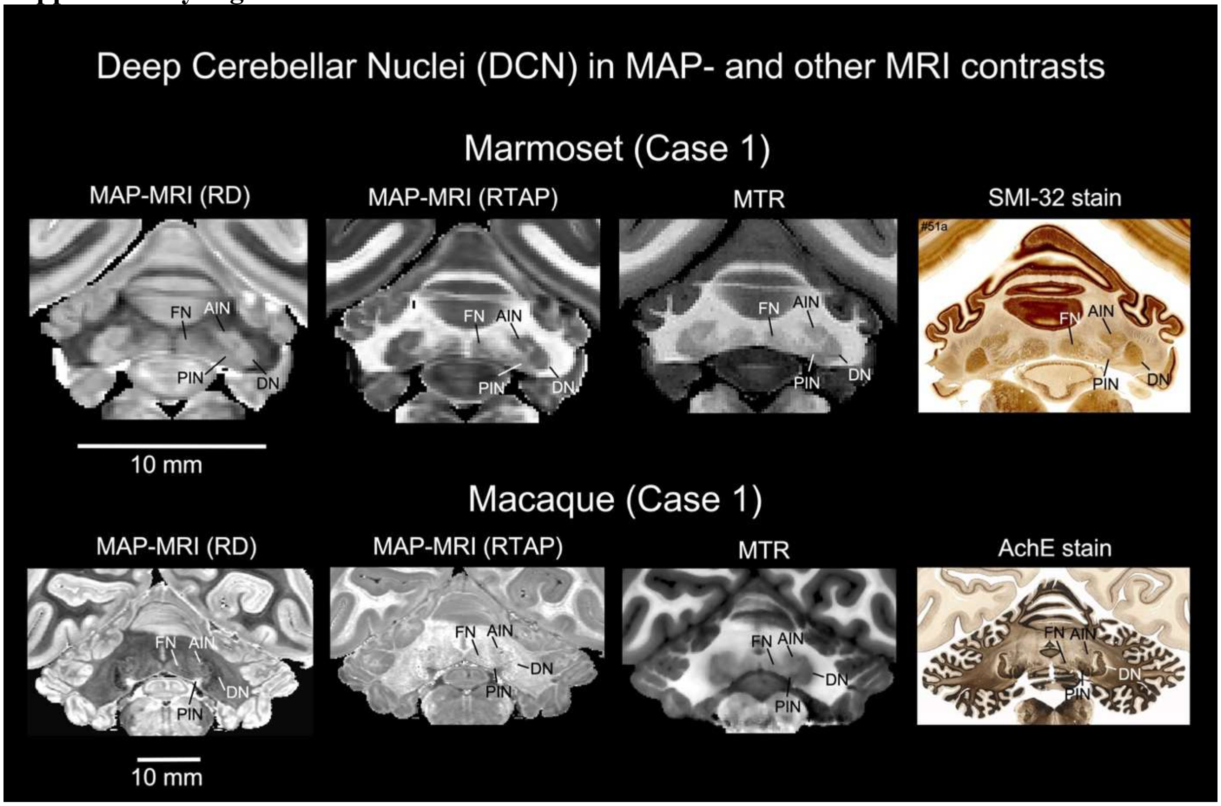


**Supplementary Fig. 1 | Multimodal MRI contrasts support delineation of deep cerebellar nuclei** Deep cerebellar nuclei (DCN): dentate nucleus (DN), anterior interposed nucleus (AIN/emboliform nucleus, EN), posterior interposed nucleus (PIN/globose nucleus, GN), and fastigial nucleus (FN), visualized using MAP-MRI-derived metrics (radial diffusivity [RD], return-to-axis probability [RTAP]) and magnetization transfer ratio (MTR) in marmoset and macaque. Matched histological sections (SMI-32 in marmoset; acetylcholinesterase [AChE] in macaque) are shown for reference. In marmosets, RD, RTAP, and MTR provide clear nuclear differentiation, whereas in macaques, MTR retains stronger contrast than diffusion-derived metrics. Across modalities, MRI-derived contrasts show consistent but variable sensitivity to DCN cytoarchitecture, supporting robustness of multimodal nuclear delineation.

**Supplementary Fig. 2**

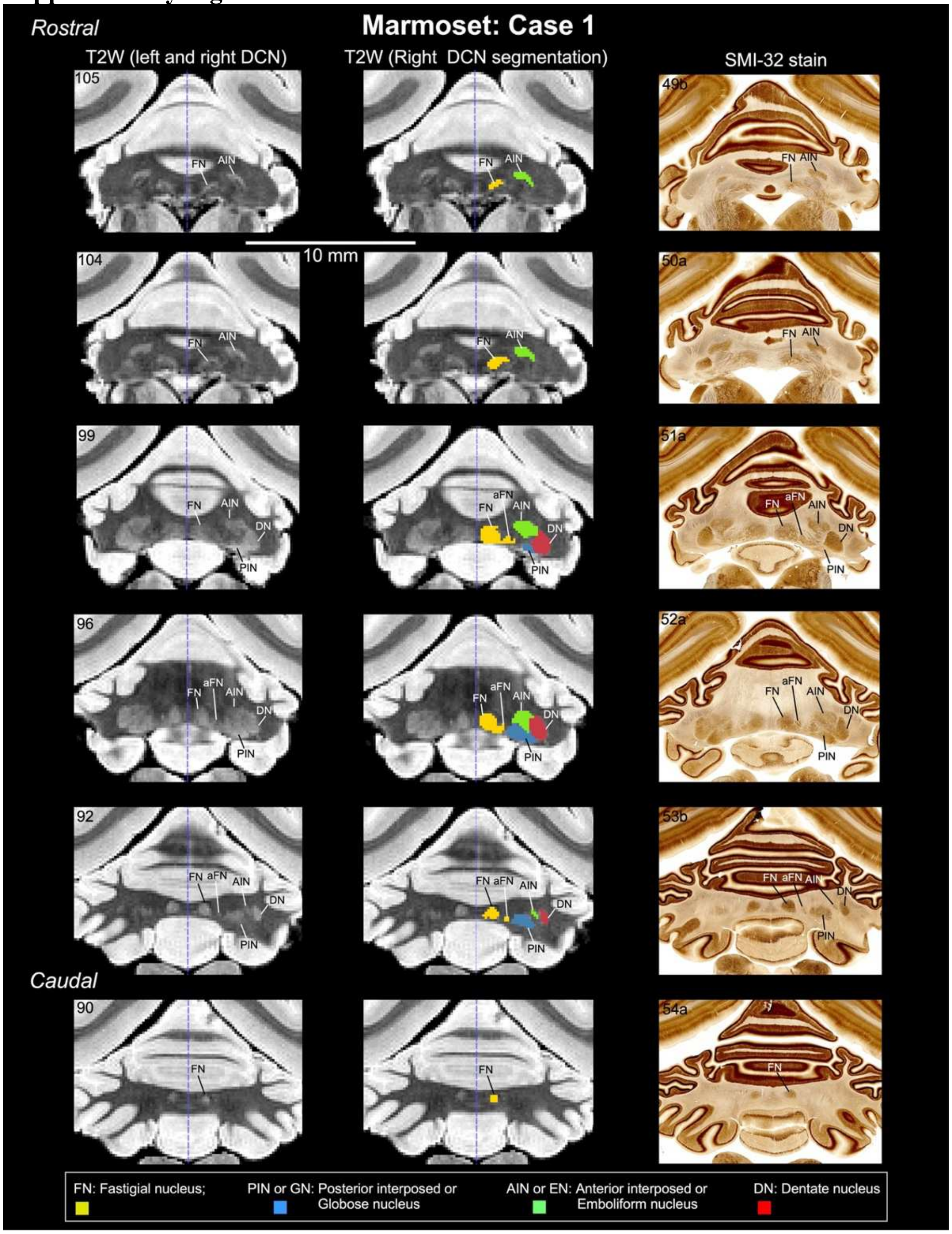


**Supplementary Fig. 2| Continuous rostrocaudal segmentation of deep cerebellar nuclei in marmoset (case 1).** Rostrocaudal T2-weighted MRI slices (150 μm isotropic resolution) of the marmoset cerebellum illustrating the full longitudinal extent of the deep cerebellar nuclei (DCN), including the dentate nucleus (DN), anterior interposed nucleus (AIN/emboliform nucleus, EN), posterior interposed nucleus (PIN/globose nucleus, GN), and fastigial nucleus (FN). Corresponding segmentation of DCN subregions is shown across the entire rostrocaudal axis (middle column). Representative histological sections stained with SMI-32 (right column) are included for key anatomical levels to confirm local correspondence between MRI-derived segmentation and cytoarchitectonic boundaries. Together, these data demonstrate

the continuity and spatial coherence of DCN segmentation across the full rostrocaudal extent of the nucleus.

**Supplementary Fig. 3**

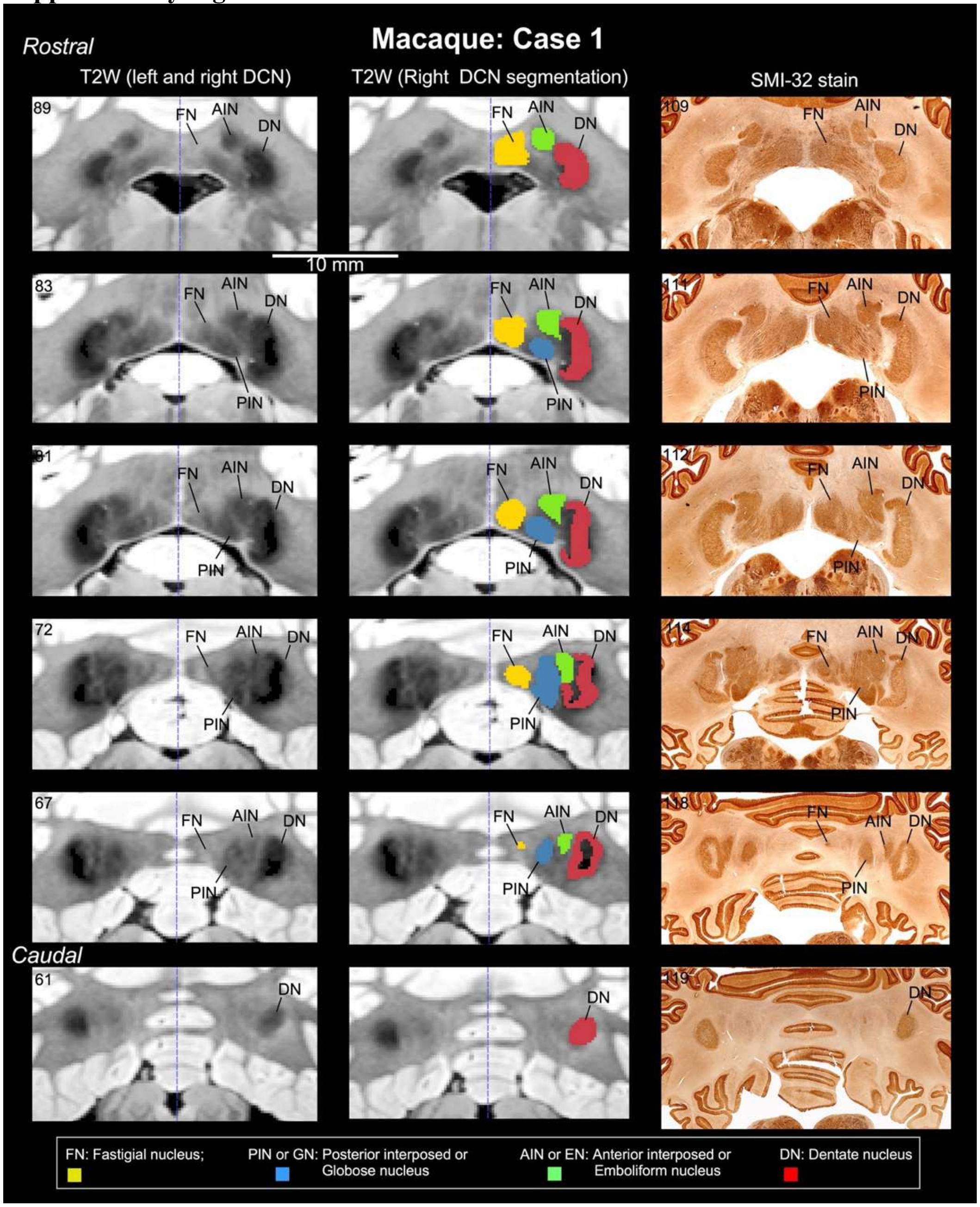


**Supplementary Fig. 3 | Histology-guided segmentation of deep cerebellar nuclei in macaque (case 1)**
Rostrocaudal T2-weighted MRI slices (200 µm isotropic resolution) of the macaque cerebellum showing the left and right deep cerebellar nuclei (DCN) with hypointense signal (left column). Corresponding segmentation of DCN subregions is shown in the right hemisphere (middle column), including the dentate nucleus (DN), anterior interposed nucleus (AIN/emboliform nucleus, EN), posterior interposed nucleus (PIN/globose nucleus, GN), and fastigial nucleus (FN). Segmentation boundaries were validated against matched histological sections from the same specimen stained with SMI-32 (right column), confirming

correspondence between MRI-defined nuclear borders and cytoarchitectonic organization. Despite species differences in MRI contrast relative to marmoset, DCN subregions exhibit conserved spatial organization and consistent anatomical delineation across modalities.

**Supplementary Fig. 4**

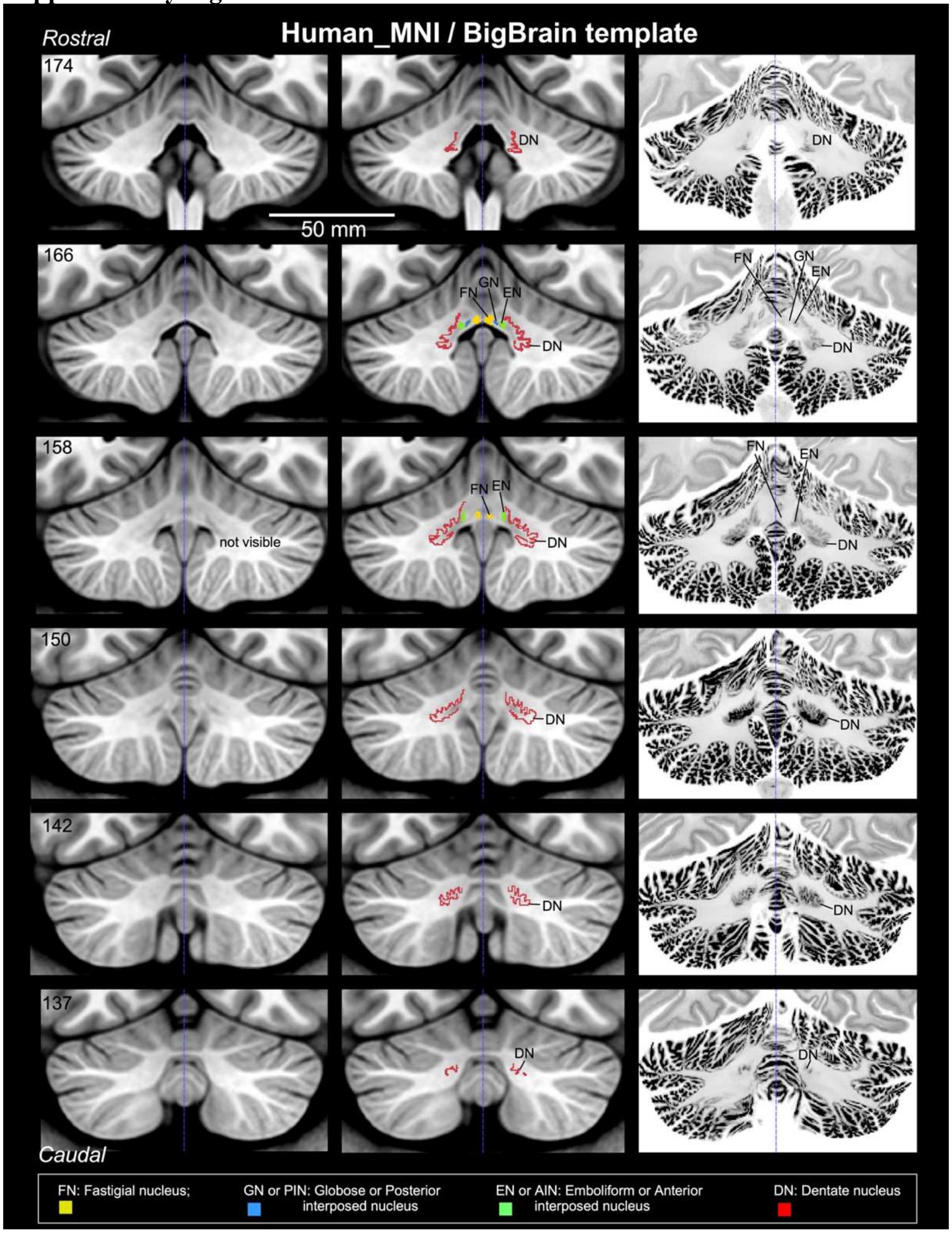


**Supplementary Fig. 4 | Histology-informed segmentation of deep cerebellar nuclei in humans**
Rostrocaudal T1-weighted MRI slices from the ICBM-152 MNI template (500 µm isotropic resolution) do not provide sufficient contrast within cerebellar white matter to reliably delineate deep cerebellar nucleus (DCN) subregions (left column), in contrast to the clear nuclear boundaries observed in nonhuman primates using T2-weighted MRI. Segmentation of DCN subregions in the right hemisphere is

shown in the middle column, including the dentate nucleus (DN), anterior interposed nucleus (AIN/emboliform nucleus, EN), posterior interposed nucleus (PIN/globose nucleus, GN), and fastigial nucleus (FN). These boundaries were defined using histology-informed priors derived from the BigBrain dataset, which was registered to the MNI (ICBM-152) template to ensure spatial correspondence between histological sections and MRI space. Corresponding cytoarchitectonic sections from the BigBrain template stained with Nissl (right column) provide anatomical reference for nuclear boundaries, enabling consistent delineation of DCN subregions despite limited MRI contrast. This approach extends the histology-validated DCN framework from nonhuman primates to humans and supports cross-species comparison of cerebellar output structures.

**Supplementary Fig. 5**

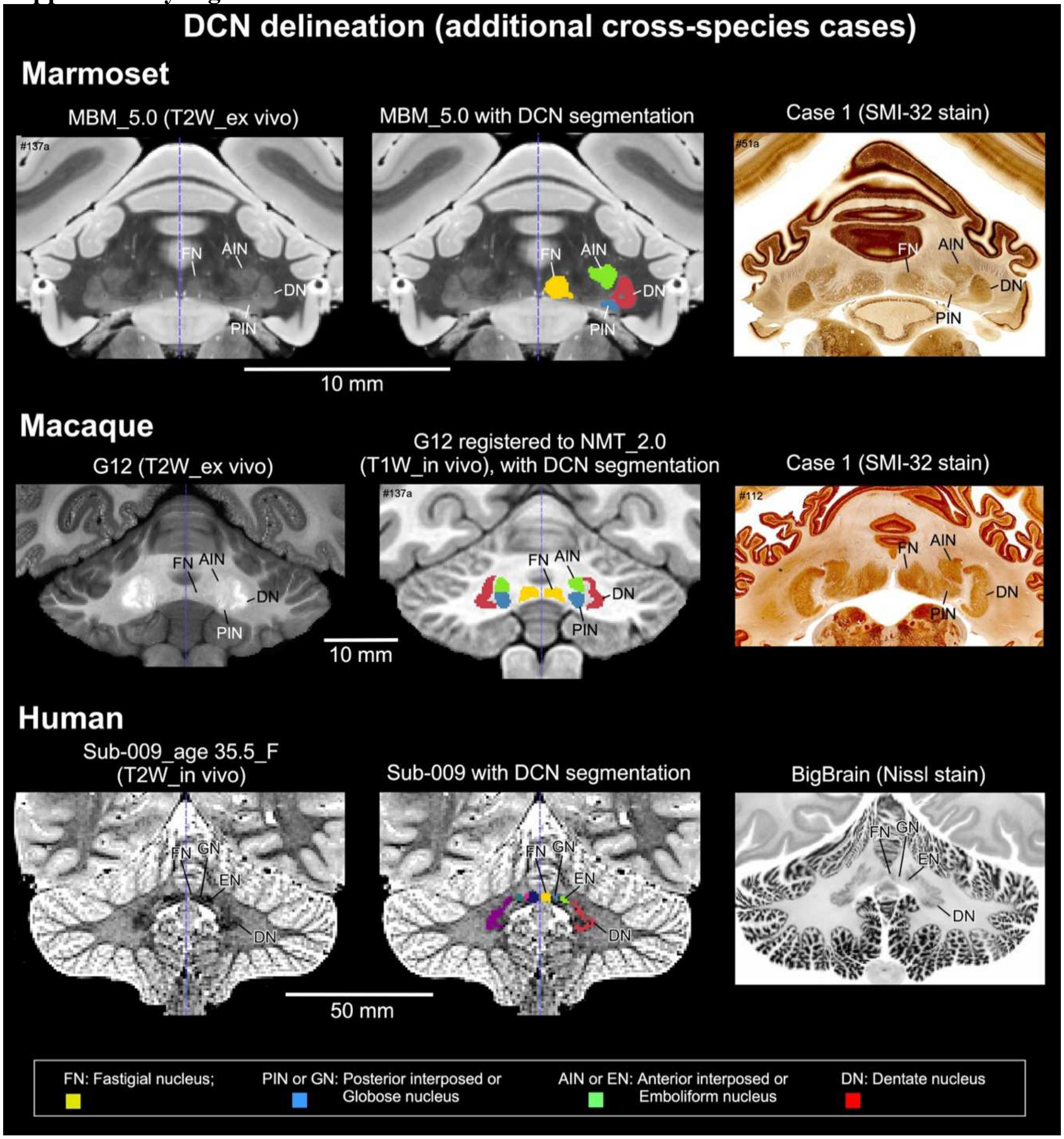


**Supplementary Fig. 5 | Cross-dataset validation of deep cerebellar nuclei segmentation across primates**

To support cross-species volumetric comparisons of the deep cerebellar nuclei (DCN), segmentation was performed in additional independently acquired datasets from marmosets, macaques, and humans, yielding five cases per species (see Fig. 4, top panel; Materials and Methods). **Top row (marmoset):** High-resolution ex vivo T2-weighted MRI (80 µm isotropic) shows hyperintense DCN subregions with segmentation in the right hemisphere. Boundaries were verified using SMI-32–stained histological sections from an independent specimen. **Middle row (macaque):** Ex vivo T2-weighted MRI (contrast-adjusted) registered to a population-based template demonstrates bilateral DCN segmentation. Nuclear boundaries were confirmed using matched SMI-32 histology from an independent case. **Bottom row (human):** In vivo T2-weighted MRI (800 µm isotropic) shows hypointense DCN signal with bilateral segmentation. Boundaries were validated using corresponding BigBrain histological sections registered to MRI space. Across species and imaging platforms, DCN subregions exhibit consistent anatomical delineation, supporting the robustness and reproducibility of segmentation used for quantitative analyses.

**Supplementary Fig. 6**

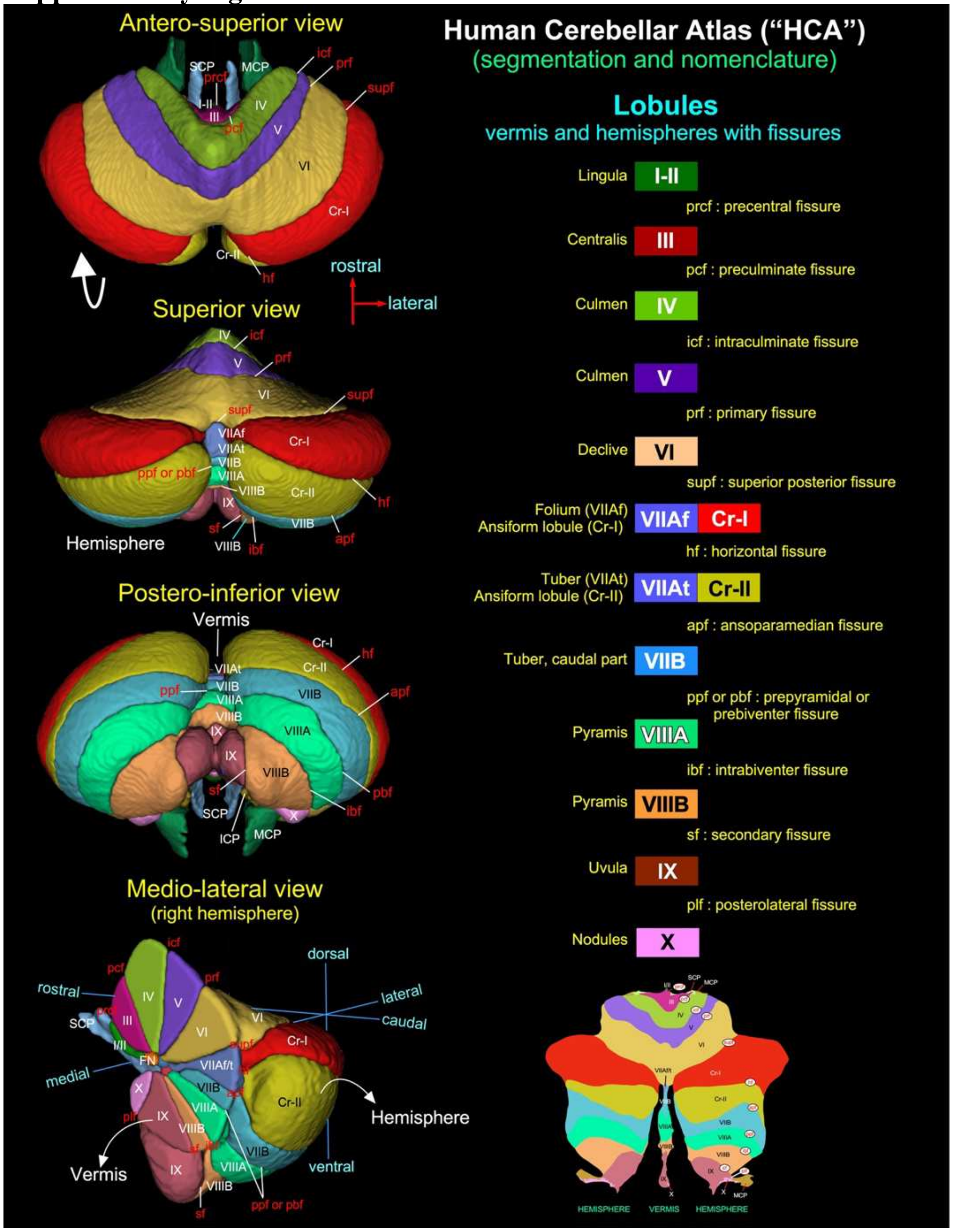

**Supplementary Fig. 6.** The HCA provides a detailed three-dimensional asymmetric segmentation of the human cerebellum, including vermis and left and right hemispheric lobules, visualized from multiple angles. It incorporates detailed fissural and lobular definitions following Schmahmann et al. [23] and includes a flat map representation with asymmetric parcellation derived from HCA. The flat map (SUIT) cerebellar template is based on the work of Diedrichsen and colleagues [62]. Together, these resources enable precise characterization of human cerebellar lobular boundaries while preserving species-specific morphology, and support systematic cross-species comparisons across primates (Fig. 5A-C).

**Supplementary Fig. 7**

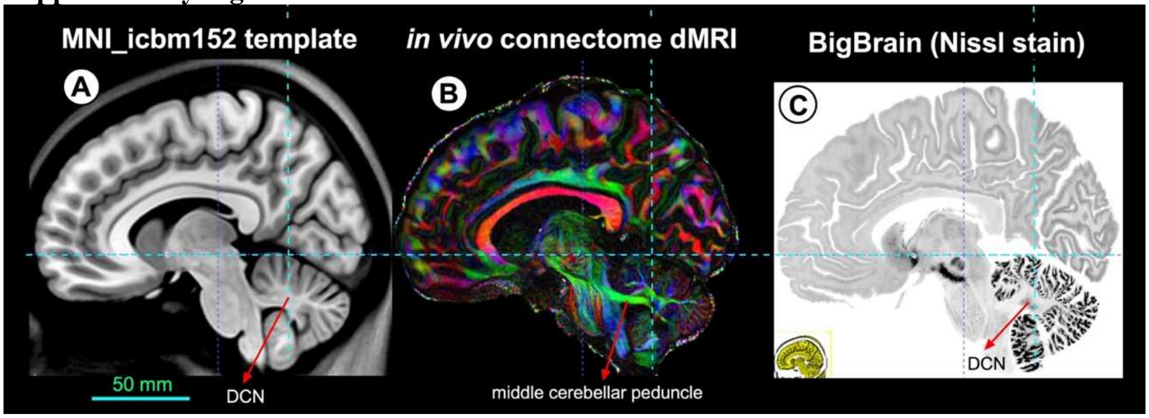


**Supplementary Fig. 7. Multimodal integration for generation of the Human Cerebellar Atlas (HCA).**
Because the deep cerebellar nuclei (DCN) are not reliably delineated in standard T1-weighted MNI templates (A), high-resolution cytoarchitectonic data from the BigBrain dataset (C) were co-registered to MNI space to refine nuclear boundaries. In parallel, in vivo diffusion MRI data from the Human Connectome Project, including direction-encoded color (DEC) volumes (B), were nonlinearly registered to the same space to delineate cerebellar peduncles and intracerebellar fiber orientations. The integration of structural MRI, histology, and diffusion data enabled precise definition of DCN subregions and white matter architecture, forming the basis of the Human Cerebellar Atlas (HCA).

## Supplementary Table 1

| Cerebellar lobules | Volume (Right cerebellum) | Volume (Left cerebellum) | Volume (total; Mean) | stdev |
|---|---|---|---|---|
| I/II | 42.4903 | 68.356 | 110.8463 | 0.0971437 |
| III | 668.593 | 714.634 | 1383.227 | 0.0986591 |
| IV | 3113.95 | 2793.49 | 5907.44 | 0.117828 |
| V | 2676.21 | 4425.09 | 7101.3 | 0.0687519 |
| VI | 8756.92 | 7831.44 | 16588.36 | 0.0870774 |
| VIIA-f/t | 160.295 | 308.902 | 469.197 | 0.0605252 |
| VIIB | 6160.48 | 6037.32 | 12197.8 | 0.10656 |
| VIIIA | 5369.54 | 5878.55 | 11248.09 | 0.110855 |
| VIIIB | 4486.22 | 5004.08 | 9490.3 | 0.116704 |
| IX | 3336.7 | 3909.5 | 7246.2 | 0.0797186 |
| X | 558.121 | 633.77 | 1191.891 | 0.0917725 |
| Cr_I | 15549.5 | 15095.5 | 30645 | 0.0656142 |
| Cr_II | 8871.5 | 9071.29 | 17942.79 | 0.100844 |
| | | | | |
| **Total number of subjects: 134** | | | | |
| **Age range: 12-52 (M/F)** | | | | |

**Supplementary Table 1 | Cerebellar lobular volumes in a normative cohort (n = 134)**

Mean gray matter volumes ($mm^3$) for cerebellar lobules I–X are reported for left and right hemispheres and as combined total volumes in 134 healthy participants (ages 12–52 years; both sexes). Lobules are organized into anterior (I–V), posterior (VI–IX), and flocculonodular (X) divisions, with detailed posterior subdivisions (VIIA–f/t, VIIB, VIIIA, VIIIB, Crus I/II) included. Interindividual variability is reported as standard deviation. Consistent with atlas-derived estimates (Fig. 6A), posterior lobules (VI–IX) and Crus I/II exhibit the largest volumes, whereas anterior lobules (I–V) and lobule X are comparatively smaller. Data were aggregated from multiple publicly available datasets (Frontiers-QC, ABIDE1, ABIDE2, and OpenNeuro).
